# Effects of sphingosine and sphingosine analogues on the free radical production by stimulated neutrophils: ESR and chemiluminescence studies

**DOI:** 10.1080/09629359791460

**Published:** 1997-12

**Authors:** A. Mouithys-Mickalad, G. Deby-Dupont, M. Hoebeke, M. Mathy-Hartert, M. Lamy, C. Deby

**Affiliations:** 1 Centre for the Biochemistry of Oxygen University of Liège Domaine Universitaire du Sart Tilman Liège 4000 Belgium; 2 Department of Anesthesia and Intensive Care Medicine University Hospital of Liège Domaine Universitaire du Sart Tilman Liège 4000 Belgium; 3 Laboratory of Experimental Physics University of Liège Domaine Universitaire du Sart Tilman Liège 4000 Belgium

## Abstract

Sphingolipids inhibit the activation of the neutrophil (PMN) NADPH oxidase by protein kinase C pathway. By electron spin resonance spectroscopy (ESR) and chemiluminescence (CL), we studied the effects of sphingosine (SPN) and ceramide analogues on phorbol 12-myristate 13-acetate (PMA, 5 × 10^-7^M) stimulated PMN (6 × 10^6^ cells). By ESR with spin trapping (100 mM DMPO: 5,5-dimethyl-1-pyrroline-Noxide), we showed that SPN (5 to 8 × 10^-6^M), C_2_-ceramide (N-acetyl SPN) and C_6_-ceramide (N-hexanoyl SPN) at the final concentration of 2 × 10^-5^ and 2 × 10^-4^M inhibit the production of free radicals by stimulated PMN. The ESR spectrum of stimulated PMN was that of DMPO-superoxide anion spin adduct. Inhibition by 5 × 10^-6^M SPN was equivalent to that of 30 U/ml SOD. SPN (5 to 8 × 10^-6^M) has no effect on *in vitro* systems generating superoxide anion (xanthine 50 mM/xanthine oxidase 110 mU/ml) or hydroxyl radical (Fenton reaction: 88 mM H_2_O_2_, 0.01 mM Fe^2+^ and 0.01 mM EDTA). SPN and N-acetyl SPN also inhibited the CL of PMA stimulated PMN in a dose dependent manner (from 2 × 10^-6^ to 10^-5^M), but N-hexanoyl SPN was less active (from 2 × 10^-5^ to 2 × 10^-4^M). These effects were compared with those of known PMN inhibitors, superoxide dismutase, catalase and azide. SPN was a better inhibitor compared with these agents. The complete inhibition by SPN of ESR signal and CL of stimulated PMN confirms that this compound or one of its metabolites act at the level of NADPH-oxidase, the key enzyme responsible for production of oxygen-derived free radicals.


Mediators of Inflammation, 6, 327–333 (1997)

### Effects of sphingosine and sphingosine analogues on the free radical production by stimulated neutrophils: ESR and chemiluminescence studies

A. Mouithys-Mickalad[1,CA], G. Deby-Dupont[1,2], M. Hoebeke[3], M. Mathy-Hartert[1], M. Lamy[1,2] and C. Deby[1]

[1]Centre for the Biochemistry of Oxygen, University of Liège, [2]Department of Anesthesia and Intensive Care Medicine, University Hospital of Liège, [3]Laboratory of Experimental Physics, University of Liège, Domaine Universitaire du Sart Tilman, 4000 Liège, Belgium

[CA]Corresponding Author
Tel: (+32) 41 66 33 60
Fax: +(32) 41 66 28 66
Email: a.mouithys⟨u416901@vm1.ulg.ac.be⟩

**Sphingolipids inhibit the activation of the neutrophil (PMN) NADPH oxidase by protein kinase C pathway. By electron spin resonance spectroscopy (ESR) and chemiluminescence (CL), we studied the effects of sphingosine (SPN) and ceramide analogues on phorbol 12-myristate 13-acetate (PMA, $5 \times 10^{-7}$ M) stimulated PMN ($6 \times 10^{6}$ cells). By ESR with spin trapping (100 mM DMPO: 5,5-dimethyl-1-pyrroline-N-oxide), we showed that SPN (5 to $8 \times 10^{-6}$ M), $C_2$-ceramide (N-acetyl SPN) and $C_6$-ceramide (N-hexanoyl SPN) at the final concentration of $2 \times 10^{-5}$ and $2 \times 10^{-4}$ M inhibit the production of free radicals by stimulated PMN. The ESR spectrum of stimulated PMN was that of DMPO-superoxide anion spin adduct. Inhibition by $5 \times 10^{-6}$ M SPN was equivalent to that of 30 U/ml SOD. SPN (5 to $8 \times 10^{-6}$ M) has no effect on *in vitro* systems generating superoxide anion (xanthine 50 mM/xanthine oxidase 110 mU/ml) or hydroxyl radical (Fenton reaction: 88 mM $H_2O_2$, 0.01 mM $Fe^{2+}$ and 0.01 mM EDTA). SPN and N-acetyl SPN also inhibited the CL of PMA stimulated PMN in a dose dependent manner (from $2 \times 10^{-6}$ to $10^{-5}$ M), but N-hexanoyl SPN was less active (from $2 \times 10^{-5}$ to $2 \times 10^{-4}$ M). These effects were compared with those of known PMN inhibitors, superoxide dismutase, catalase and azide. SPN was a better inhibitor compared with these agents. The complete inhibition by SPN of ESR signal and CL of stimulated PMN confirms that this compound or one of its metabolites act at the level of NADPH-oxidase, the key enzyme responsible for production of oxygen-derived free radicals.**

**Key words:** Sphingosine, Ceramides, Active oxygen species, Polymorphonuclear neutrophil, Chemiluminescence, Electron spin resonance

### Introduction

The production of active forms of oxygen by PMN stimulated by PMA or other activators has been demonstrated by spectrophotometric techniques, by measurement of oxygen consumption, and by chemiluminescence (CL).[1–8] The enzymatic activities of the NADPH oxidase complex and the granular myeloperoxidase are the main factors responsible for this active oxygen species and chemiluminescence production,[9] but it has been suggested that xanthine oxidase would also participate in this production.[10,11]

Sphingosine (SPN) and some of its derivatives inhibit this production of active oxygen species by PMA stimulated PMN,[12,13] probably by acting at protein kinase C, a key effector in PMA stimulatory action.[13] It was also suggested that SPN and its phosphorylated derivatives, when added to PMA-stimulated PMNs, can modulate intracellular levels of calcium,[14] thus acting as second messenger. Other derivatives of SPN, such as ceramides ($C_2$ ceramide, or N-acetyl-sphingosine, and $C_6$ ceramide, or N-hexanoyl-sphingosine), can also modify cellular activation and calcium homeostasis in PMN.[15] If the inhibitory effect of sphingolipids was considered as due to a physical interaction between the NADPH-oxidase components and the sphingolipid-ceramides as hypothesized by Bazzi and Nelsestuen,[16] several works however have reported that SPN and ceramides could induce apoptosis[17] and are involved in the regulation of cell growth and differentiation.[18] More recently, Augé *et al.*[19] observed that sphingomyelin ceramide signalling pathway was involved in oxidation of LDL-induced cell proliferation.

© 1997 Rapid Science Publishers

Thus, although numerous studies on the role of sphingomyelin ceramides have been performed, it seems that some questions remain to be elucidated as to the exact way of action of these sphingolipids.

SPN and related compounds thus appear as specific inhibitors of NADPH-oxidase activity, but their inhibiting effects were never demonstrated by electron spin resonance (ESR) techniques.[12–20] ESR technique, associated with spin trapping, allows the direct study of the free radicals produced during the respiratory burst of PMN.[21,22] The superoxide anion ($O_2^{\cdot -}$) and hydroxyl radical ($HO^{\cdot}$), which have a very short lifetime, can be detected using a nitrone compound, 5,5-dimethyl-1-pyrroline-N-oxide (DMPO) as spin trap.[23] The corresponding DMPO-$^{\cdot}$OOH and DMPO-$^{\cdot}$OH spin adducts are more stable and possess a well-known hyperfine structure.[24,25]

The aim of this work was to demonstrate by ESR the effects of SPN, N-acetyl SPN and N-hexanoyl SPN on the production of free radicals by PMA stimulated PMN. These original ESR studies were confirmed by CL studies and compared with known inhibitors (superoxide dismutase, catalyse and azide) of the stimulated PMN.

### Materials and Methods

### Reagents

D-sphingosine, N-acetyl sphingosine and N-hexanoyl sphingosine were obtained from bovine brain cerebrosides (Sigma). Ficoll solution was prepared with 57 g/l Ficoll 400 (Sigma), 90 g/l sodium diatrizoate (Sigma) and 0.1% ethylenediaminetetra-acetic acid (EDTA, analytical grade, Merck). Xanthine-oxidase was from cow milk (Boehringer Mannheim). Catalase (from bovine liver) and superoxide dismutase (SOD from bovine erythrocytes) were from Sigma. Xanthine, alun $[(NH_4)_2Fe(SO_4)_2{\cdot}6H_2O]$, EDTA and hydrogen peroxide ($H_2O_2$) were from Merck (analytical grade). DMPO was from Aldrich and purified with activated charcoal as described by Green and Hill.[26] PMA was obtained from Sigma, dissolved in dimethylsulphoxide (DMSO) and stored at −20°C until use. Ethanol was from UCB (analytical grade). Luminol (3-aminophtalhydrazide) was from Aldrich. Phosphate buffer contained: NaCl (8 g/l), KCl (0.2 g/l), $Na_2HPO_4$ (1.15 g/l) and $KH_2PO_4$ (0.2 g/l). Hank's balanced salt solution (HBSS) contained $CaCl_2{\cdot}2H_2O$ (0.185 g/l), KCl (0.40 g/l), $MgCl_2{\cdot}6H_2O$ (0.10 g/l), $MgSO_4{\cdot}7H_2O$ (0.10 g/l), $KH_2PO_4$ (0.06 g/l), NaCl (8 g/l), $NaHCO_3$ (0.35 g/l), $Na_2HPO_4$ (0.48 g/l) and glucose (1 g/l).

### Neutrophil isolation and activation

Blood was obtained from normal healthy volunteers. After centrifugation, plasma was discarded. The buffy coat was diluted (2:1) with Ficoll solution.[27] After 20 min centrifugation at 450 g (20°C), the supernatant was diluted (2:1) with 0.9% NaCl and further centrifuged for 20 min at 1250 g (20°C). The precipitate was treated with a hypotonic solution ($NH_4Cl$ 0.15 mM; EDTA 0.1 mM; $NaHCO_3$ 10 mM) to destroy the remaining erythrocytes. Neutrophils were then suspended in HBSS, counted and kept at room temperature. SPN, N-acetyl SPN and N-hexanoyl SPN were dissolved in ethanol; these compounds were preincubated with neutrophils for 8 min at room temperature prior to addition of the activator (PMA).

### *In vitro* production of free radicals

Superoxide anion was generated by the xanthine (50 mM), xanthine oxidase (110 mU/ml) system, and $HO^{\cdot}$ by the Fenton reaction: $H_2O_2$ (88 mM), $Fe^{2+}$ (0.01 mM) and EDTA (0.01 mM), in aqueous solution or HBSS.

### ESR experiments

The inhibition by SPN and analogues of the production of free radicals by $5 \times 10^{-7}$ M PMA activated PMN ($6 \times 10^6$ cells/ml) was examined using spin trapping technique, and was compared with the effect of other classical inhibitors such as SOD (which inactivates the superoxide anion) and catalase (which inactivates hydrogen peroxide). The potential scavenger effect of SPN on $O_2^{\cdot -}$ and $^{\cdot}OH$ was also investigated by direct addition of SPN to the $O_2^{\cdot -}$ or $^{\cdot}OH$ generating systems. After preparation, the mixture was immediately transferred to a flat cell and placed in the $TM_{110}$ cavity of the ESR spectrometer. The spectra were recorded at room temperature on a Bruker ESP 300 E spectrometer operating at X-band (9.76 GHz) with 100 kHz modulation frequency and 3475 G magnetic field. These measurements were performed with nonsaturating 20 mW microwave power, modulation amplitude 1.01 G, scan range 100 G, and receiver gain $2 \times 10^4$. The spin trapping studies were performed by using DMPO as a spin trap at a final concentration of 100 mM, and a final volume of 0.8 ml HBSS.

### CL assays

CL was measured with a Bio-Orbit 1251 luminometer following the technique of Easmon *et al.*[28] Luminol ($10^{-4}$ M), the PMN suspension ($2{\cdot}5 \times 10^5$ cells/ml), the respiratory burst activator (PMA $8 \times 10^{-7}$ M), and the tested compound (SPN, N-acetyl SPN and N-hexanoyl SPN) at final concentrations ranging from $2 \times 10^{-6}$ to $10^{-4}$ M in phosphate buffer saline at pH 7.4 were sequentially added to a circular polystyrene reaction vessel (final reaction volume: 0.5 ml). CL was followed at 37°C for 10 min. The data were recorded and digitized, and the CL values were expressed in arbitrary units. Well-characterized inhibitors of the production of activated oxygen species were tested: SOD (100–300 U/ml) for $O_2^{\cdot-}$, catalase (200 mg/ml) for $H_2O_2$, and azide ($10^{-6}$ to $10^{-4}$ M) for inhibition of myeloperoxidase.

### Results

### ESR studies

*ESR spectra of activated PMNs.* When PMN were stimulated with PMA in the presence of DMPO, the resulting ESR spectrum was characterized by a signal mixture that can be attributed to signals of DMPO-˙OOH ($a_N = 14{\cdot}3$ G, $a_H = 1{\cdot}3$ G) adduct and DMPO-˙OH ($a_H = a_N = 14{\cdot}87$ G) adduct[29,30] (Fig. 1A). However, the presence of the signal of DMPO-˙OH spin adduct can be the result of DMPO-˙OOH decomposition instead of ˙OH trapping.[29,30]

Indeed, we noted that over time, the ESR signal amplitude of DMPO-˙OOH decreased while that of DMPO-˙OH was increased. Addition of ethanol to activated PMN, at the final concentration of 1% (Fig. 1E), does not change the spectrum observed. Amplitude of the ESR signal of superoxide anion adduct depended on time and of the state of neutrophils. However, when neutrophils were unstimulated with PMA, no ESR signal of DMPO spin adducts was seen (Fig. 1B). The ESR spectrum C of Fig. 1 was obtained when neutrophils were killed by incubation at 45°C for 30 min before addition of PMA and DMPO. Here again, no ESR signals were formed.

*Effects of SPN and analogues.* Concentrations of $8 \times 10^{-6}$ M of SPN led to inhibition of PMA-activated PMN, resulting in the disappearance of ESR signals (Fig. 1D). The inhibition of ESR signals by SPN was dose-dependent and complete for $5 \times 10^{-6}$ M SPN (Fig. 2). The two other ceramides also inhibited the ESR signal in a dose-dependent manner, but they were less active than SPN (Fig. 3).

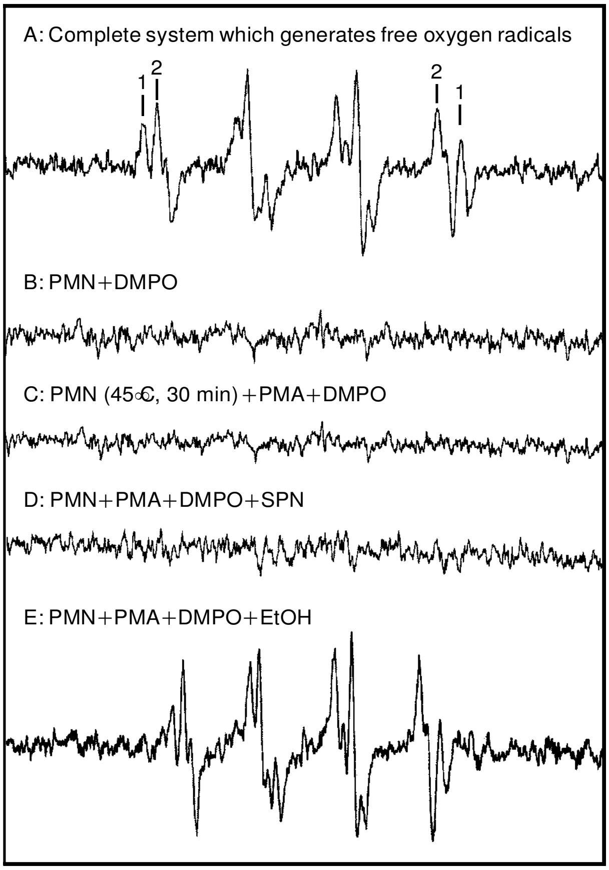

FIG. 1. ESR spectra from neutrophils ($6 \times 10^6$ cells/ml) stimulated with PMA ($5 \times 10^{-7}$ M). For ESR conditions, see text. Scan A: complete system which generates oxygen free radicals (1: DMPO-˙OH adduct; 2: DMPO-˙OOH adduct). Scan B: same as A without PMA. Scan C: same as A after destruction of the cells by incubation at 45°C for 30 min before addition of PMA. Scan D: same as A + sphingosine $8 \times 10^{-6}$ M. Scan E: same as A + 1% ethanol. The hyperfine splittings for scan A were: $a_H = 14{\cdot}3$ G and $a_N = 14{\cdot}87$ G.

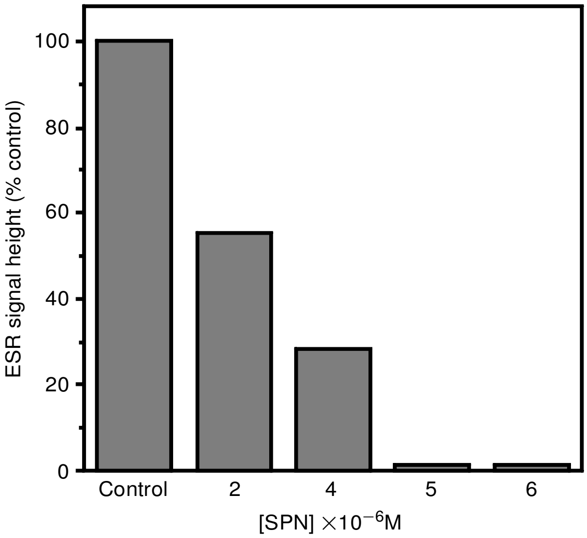

FIG. 2. Effect of increasing concentrations of sphingosine on ESR signal heights of activated human neutrophils ($6 \times 10^6$ cells/ml). Spin trap (DMPO, 100 mM), activator PMA ($5 \times 10^{-7}$ M).

*In vitro production of $O_2^{\cdot-}$ and $HO^{\cdot}$.* While Fig. 4 suggests that SPN (dissolved in ethanol) inhibits the signal of DMPO-˙OH adduct production by the Fenton reaction, however, when ethanol was used alone (at 0.25% and 0.5%) the concentrations respectively present in the $2\times10^{-6}$ and $4\times10^{-6}$ M SPN solutions), a similar or even more pronounced inhibiting effect was observed (Fig. 4). As shown in Fig. 5, the ESR spectrum of DMPO-˙OH adduct was modified by ethanol (1%) (Fig. 5b) compared with the control signal (Fig. 5a). This modified spectrum corresponds to a DMPO-ethoxy spin

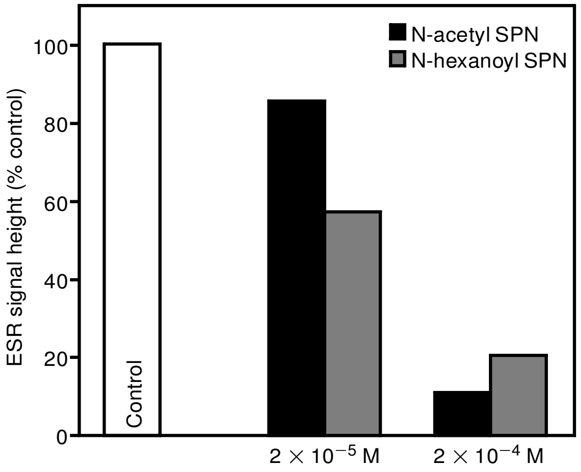

FIG. 3. Effects of two sphingosine analogues (N-acetyl SPN and N-hexanoyl SPN) on free radicals produced by stimulated-PMN ($6\times10^{6}$ cells/ml). Spin trap (DMPO (100 mM), activator PMA ($5\times10^{-7}$ M).

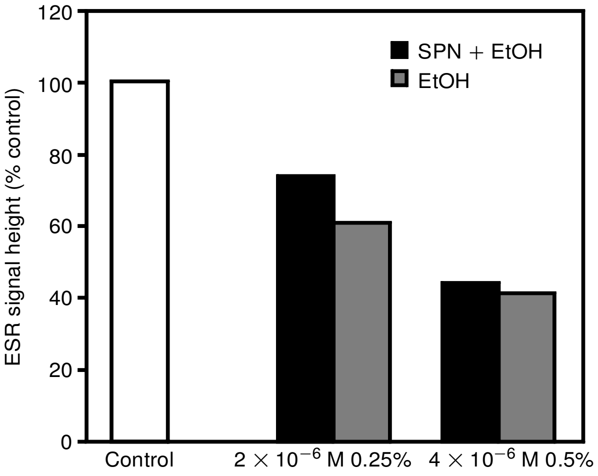

FIG. 4. The effects of sphingosine dissolved in ethanol (EtOH) and ethanol alone on ESR signal heights (DMPO 100 mM) by hydroxyl radicals generated by the Fenton reaction. [$H_2O_2$ (88 mM), $Fe^{2+}$ (0.01 mM), EDTA (0.01 mM).] Sphingosine was added at two concentrations (2 and $4\times10^{-6}$ M). Ethanol was used at 0.25 and 0.5%.

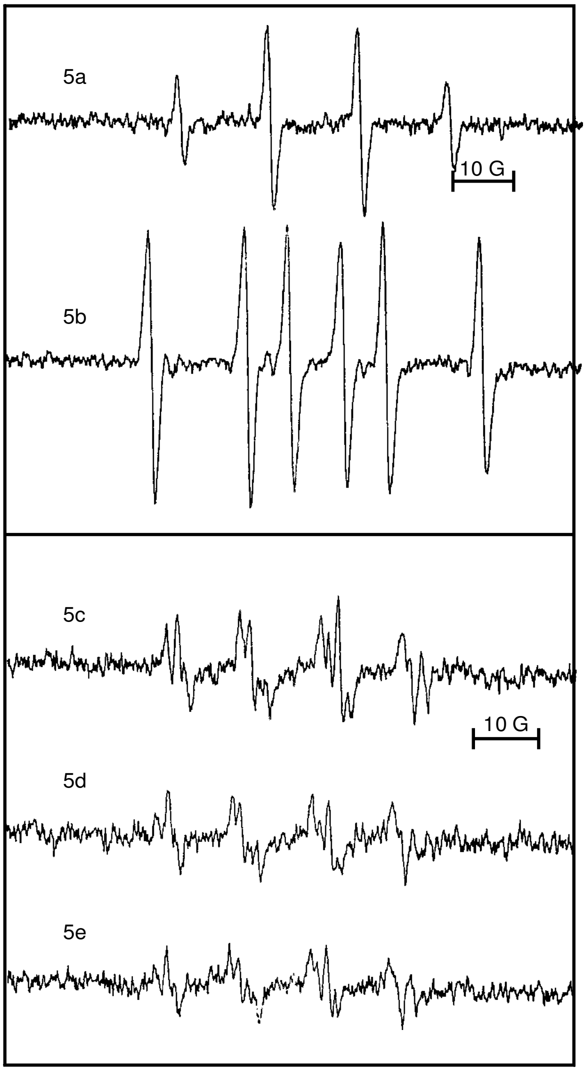

FIG. 5. ESR spectra (DMPO 100 mM) of the spin adduct of hydroxyl radical generated by the Fenton reaction [$H_2O_2$ (88 mM), $Fe^{2+}$ (0.01 mM), EDTA (0.01 mM) (a and b) and superoxide spin adduct generated by xanthine (50 mM)/xanthine-oxidase (110 mU/ml) system in HBSS buffer pH 7.4 (c, d and e). (a) Normal spectrum (hyperfine splittings for DMPO-˙OH: $a_H = a_N = 14{\cdot}87$ G). (b) Same as 5a + 1% ethanol: the spectrum of the DMPO-ethoxy adduct is observed (hyperfine splittings for DMPO-˙C($CH_3$)OH: $a_H = 15{\cdot}67$ G, $a_N = 23{\cdot}0$ G). (c) Complete system xanthine/xanthine-oxidase generating superoxide radical, in the presence of DMPO, without SPN. (d) Complete system in the presence of $4\times10^{-6}$ M SPN. (e) Complete system in the presence of $8\times10^{-6}$ M SPN.

adduct. Indeed, hydroxyl radical reacts with ethanol to produce α-hydroxylethyl radicals. These secondary radicals can then react with the spin trap to produce an adduct with an ESR spectrum distinguishable from that of the hydroxyl adduct as previously shown.[31,32] We conclude that despite of the high concentrations (5 to $8\times10^{-6}$ M) used, SPN does not act on the hydroxyl radical; the apparent decrease of the ESR signal is due to a secondary reaction of $^{\cdot}OH$ radicals with ethanol solvent. The ESR spectrum of the hydroxyl adduct with DMPO is composed of a quartet with a characteristic pattern of intensities (1:2:2:1), whose hyperfine structure shows the following coupling constants $a_N = a_H = 14{\cdot}87$ G, while those of hydroxylethyl adduct are $a_N = 15{\cdot}67$ G and $a_H = 23{\cdot}0$ G.

As far as $O_2^{\cdot -}$ is concerned (produced in the acellular system xanthine/xanthine-oxidase, Fig. 5c), SPN has no effect (Fig. 5d and 5e); no significative changes of the ESR spectrum were observed despite concentrations of $8 \times 10^{-6}$ M SPN (Fig. 5e).

*Comparison with classical inhibitors.* SOD was inhibitory in a dose-dependent manner on ESR signals of DMPO-$^{\cdot}$OOH spin adducts (Table 1A), and low doses were sufficient to yield complete inhibition in ESR experiments. Catalase did not exert a complete inhibition even at doses of 200 mg/ml. Compared with these two 'classical' inhibitors, sphingosine was extremely active, with full inhibition seen at $8 \times 10^{-6}$ M for ESR spectra.

### CL studies

*Effects of SPN and analogues.* The CL of PMA stimulated PMN is inhibited by the three compounds in a dose-dependent manner (Fig. 6): SPN and N-acetyl SPN had similar inhibitory activities, higher than that of N-hexanoyl SPN. At $2 \times 10^{-5}$ M, the inhibition of CL activity was almost complete for SPN and N-acetyl SPN (99% inhibition). N-hexanoyl SPN was inactive at $2 \times 10^{-6}$ M and presented 82.8% at $2 \times 10^{-4}$ M.

*Comparison with classical inhibitors of PMN CL.* The data of Table 1B indicate that SPN at $10^{-4}$ M was more active than 300 U/ml SOD, 200 mg/ml catalase and $10^{-4}$ M azide. In the same way, the two SPN analogues were also more active than SOD, catalase and azide.

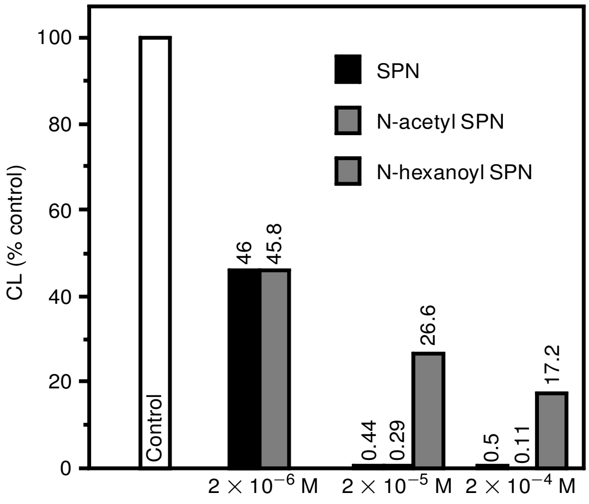

FIG. 6. Comparison of the effects of SPN analogues with those of SPN on luminol enhanced CL (in % control) of PMA-activated PMN ($2{\cdot}5 \times 10^5$ cells/ml).

### Discussion

Over the past years, the role attributed to the sphingomyelin ceramides (sphingosine and analogues) in biological systems is increasing. These compounds have even emerged as important signalling molecules involved in many cellular processes.[33] Moreover, some ceramide analogues have been also demonstrated to inhibit the respiratory burst of neutrophils induced by agonist-receptor of PMN, and to hinder the action of different modulators of PMN activity, such as tumour necrosis factor (TNF$\alpha$).[34] More recently, Fuortes *et al.*[35] reported that the brief elevation of ceramide blood concentration in response to TNF$\alpha$, could mediate the lag period observed in the response of PMN. In fact, a similar lag period was also seen with other soluble physiological agonists. Thus, among various metabolites derived from the hydrolytic activity of sphingomyelinase (SPM), the short-chain ceramide analogues such as $C_2$-ceramide and $C_6$-ceramide were often

**Table 1**. Comparison of the effects of SPN and two classical inhibitors of PMN activation on ESR signal amplitude (A) and CL activity (B)

| Added compound | ESR assay A | | CL assay B | |
|---|---|---|---|---|
| | Conc. | Signal ampl. (%) | Conc. | CL activity (%) |
| None (control) | – | 100 | – | 100 |
| Catalase | 200 mg/ml | 48 | 200 mg/ml | 100 |
| SOD | 10 U/ml | 10 | 100 U/ml | 80 |
| | 30 U/ml | 0.6 | 300 U/ml | 50 |
| Azide | $10^{-3}$ M | 65 | $10^{-4}$ M | 71 |
| Sphingosine | $5 \times 10^{-6}$ M | 0.4 | $10^{-4}$ M | 0.7 |

used to better understand their involvement in the cellular signalling mechanisms. Like sphingosine, its sphingosine 1-phosphate metabolite (SPP) mobilizes $Ca^{2+}$ by an intracellular mechanism.[14] As suggested by Hannun,[36] ceramide plays a key role as regulator of antiproliferative and apoptotic pathways and as an inhibitor of protein signalling and secretion. Thus, the study of their functions, metabolism and mechanisms of action raises a great interest. It is also known that inhibition of ceramide-mediated apoptosis by activation of protein kinase C results from stimulation of sphingosine kinase and the concomitant increase in intracellular SPP.[36]

Chao *et al.* reported that SPP was the main sphingolipid involved in the signal transduction pathway induced by the platelet-derived growth factor (PDGF) receptor stimulation in cells.[37] Several other teams have underlined the role of SPP in these various mechanisms. Although the role of sphingolipids is now widely admitted, the exact mechanism by which they act still needs to be completely elucidated. Many questions, however, remain, for example understanding whether the ceramide has an active role by itself in the intracellular signalling or if it is SPP which is the effective mediator and sphingosine as an intermediate agent only.

Previous studies (by spectrophotometric techniques or measurement of oxygen consumption) have reported that sphinganine and SPN can inhibit the production of activated species of oxygen by PMN.[12,13,38] We demonstrated here that SPN and its ceramide analogues (N-acetyl SPN and N-hexanoyl SPN) inhibit the ESR signal obtained by the reaction of DMPO with free radicals produced by the PMA stimulated PMN. The production of $O_2^{\cdot -}$ during the respiratory burst[39] is clearly demonstrated by the observation of the DMPO-$^{\cdot}$OOH spin adduct, while $^{\cdot}$OH is not generated during the experiment. Indeed, in the presence of ethanol, a well-known $^{\cdot}$OH scavenger, the DMPO-ethoxy spin adduct does not appear. The DMPO-$^{\cdot}$OH signal present in the ESR spectrum is the result of DMPO-$^{\cdot}$OOH decomposition. The spectrum we observed is consistent with that described in the literature.[29] The failure to observe organized spectra when PMN are either unstimulated or are destroyed by heat confirms that cellular activity is required for production of $O_2^{\cdot -}$ derived free radicals.

The production of superoxide anion by stimulated PMN results from activation of membrane-bound NADPH oxidase. When SPN is preincubated for 8 min with stimulated PMN, the ESR signal is totally suppressed. This inhibitory activity of SPN is dose dependent. SPN may either inhibit NADPH-oxidase, and/or may directly scavenge the activated oxygen species thus produced. Two further experiments were performed in order to determine which mechanism was responsible for the inhibition seen in our study. The first involved incubating an *in vitro* superoxide-generating system with SPN, while the second looked at the effect of SPN on *in vitro* production of the hydroxyl radical (by the Fenton reaction). The results show that SPN has no direct action on either species and has no action on the xanthine oxidase activity. Initial confrontation of these experiments seemed to imply inhibition of *in vitro* hydroxyl radical production; closer analysis showed that the inhibition was due solely to the ethanol used as a solvent for SPN. Ethanol reacts with the hydroxyl radical yielding an ethoxy radical, which is then trapped by DMPO.[31,32] The inhibition of the signal of the DMPO-$^{\cdot}$OH adduct is explained by the increase of signal of the DMPO-ethoxy adduct.

The ESR results obtained with classical inhibitors such as SOD and catalase are in agreement with data from other authors.[29,30] In comparison, SPN exerts a stronger inhibitory effect than the two classical inhibitors tested. This confirms an action at the initial step of NADPH oxidase activation. The weak effect of catalyse at high concentration may be explained by the fact that an important part of $O_2^{\cdot -}$ directly reacts with DMPO leading to spin DMPO-$^{\cdot}$OH adduct, without being transformed. For superoxide dismutase, we obtained similar results to those described in the literature.[29] Two of the ceramides tested (SPN and N-acetyl SPN) exhibited the same inhibitory effects on free radicals produced by PMA stimulated PMN, while N-hexanoyl SPN was less active.

CL assays confirmed the inhibitory effect of SPN and its analogues observed by ESR technique. Indeed, at low doses SPN and N-acetyl SPN inhibited about 50% of the CL activity and at high concentrations, CL activity was completely suppressed. Here also, N-hexanoyl SPN was less active. To explain this difference, we could have hypothesized that N-hexanoyl SPN does not correctly enter into the cell and does not interact exactly with NADPH oxidase components, due to the length of its carbon chain. These findings suggest a physiological role for sphingosine and related compounds, in particular in the downregulation of protein kinase C activity.

Our results corroborate with those described previously[12,13] and are consistent with a direct action of SPN on the activation of NADPH oxidase via protein kinase C. This naturally occurring substance differs from other known inhibitors of protein kinase C, and has been seen to exert powerful and reversible inhibition. Previous work has shown production of sphingosine from substances such as ceramides, by the action of enzymes such as N-acetylsphingosine amidohydrolases.[12] Similarly, its intracellular production by various metabolic pathways has been suggested (sphingomyelin hydrolysis). This provides a physiological role for its use as a negative regulator or modulator of protein kinase C.[38,40] We found that SPN totally suppresses the ESR signal of stimulated PMN, but is inactive on the enzymatic activity of xanthine-oxidase, and is not a direct scavenger of the free oxygen-derived radicals $O_2^{\cdot -}$ and $^{\cdot}OH$. These results confirm that the major role in the production of free radicals by stimulated PMN is played by NADPH-oxidase and not by xanthine oxidase. If xanthine oxidase would intervene in this production, we would not observe a complete disappearance of the ESR signal of DMPO-$^{\cdot}$OOH adduct with $10^{-6}$ M SPN, a specific inhibitor of NADPH-oxidase.

### References

1. Lehmeyer JE, Synderman R, Johnston Jr RR. Stimulation of neutrophil oxidative metabolism by chemotactic peptide: influence of calcium ion concentration and cytochalasin B and comparison with stimulation by phorbol myristate acetate. *Blood* 1979; **54**: 35–45.
2. Williams AJ, Cole PJ. The onset of polymorphonuclear leucocyte membrane-stimulated metabolic activity. *Immunology* 1981; **43**: 733–739.
3. Bender JG, Van Epps DE. Analysis of bimodal chemiluminescence pattern stimulated in human neutrophils by chemotactic factors. *Infect Immun* 1983; **41**: 1062–1070.
4. Roschger P, Graninger W, Klima H. Stimulation detection of native and luminol-dependent luminescence of stimulated human leukocytes. *Biochem Biophys Res Commun* 1984; **123**: 1047–1053.
5. Dahlgren C, Aniansson H, Magnusson KE. Pattern of formylmethionyl-leucyl-phenylalanine-induced luminol and lucigenin-dependent chemiluminescence in human neutrophils. *Infect Immun* 1985; **47**: 326–328.
6. Allen RC, Phagocytic leukocyte oxygenation activities and chemiluminescence: a kinetic approach to analysis. *Methods in Enzymology* 1986; **133**: 449–493.
7. Rudkowski R, Ziegler JB, Graham GG, Joulianos G. Gold complexes and activation of human polymorphonuclear leukocytes. *Biochem Pharmacol* 1992; **44**: 1091–1098.
8. James KH. Oxygen-derived toxins generated by neutrophils and their microbicidal mechanisms. In: Barton DR, Martell AE, Sawyer DT, eds. *Proceedings of the Fifth International Symposium on the Activation of Dioxygen and Homogeneous Catabolic Oxidation*. New York: Plenum Press, 1993; 267–286.
9. Lundqvist H, Follin P, Khalfan L, Dahlgren C. Phorbol-myristate acetate-induced NADPH oxidase activity in human neutrophils: only half the story has been told. *J Leukoc Biol* 1996; **59**: 270–279.
10. Tubaro E, Lotti B, Santiangelli C, Cavallo G. Xanthine oxidase: an enzyme playing a role in the killing mechanism of polymorphonuclear leucocytes. *Biochem Pharmacol* 1980; **29**: 3018–3020.
11. Tritsch GL, Niswander PW. Modulation of macrophage superoxide release by purine metabolism. *Life Sciences* 1983; **32**: 1359–1362.
12. Hannun YA, Loomis RC, Merrill AH Jr, Bell RM. Sphingosine inhibition of protein kinase C activity and phorbol dibutyrate binding in vitro and human platelets. *J Biol Chem* 1986; **261**: 12604–12609.
13. Wilson E, Rice WG, Kinkade JM Jr, Merrill AH Jr, Arnold RR, Lambeth JD. Protein kinase C inhibition by sphingoid lung chain bases: effect on secretion in human neutrophils. *Arch Biochem Biophys* 1987; **259**: 204–214.
14. Fatatis A, Miller RJ. Sphingosine and sphingosine 1-phosphate differentially modulate platelet-derived growth factor-BB-induced $Ca^{2+}$ signalling in transformed oligodendrocytes. *J Biol Chem* 1996; **271**: 295–301.
15. Wong K, Li XB, Hunchuk N. N-acetylsphingosine ($C_2$-ceramide) inhibited neutrophil superoxide formation and calcium influx. *J Biol Chem* 1995; **270**: 3056–3062.
16. Bazzi MD, Nelsestuen GL. Mechanism of protein kinase C inhibition by sphingosine. *Biochem Biophys Res Comm* 1987; **146**: 203–207.
17. Zhang P, Liu B, Jenkins GM, Hannun YA, Obeid LM. Expression of neutral sphingomyelinase identifies a distinct pool of sphingomyelin involved in apoptosis. *J Biol Chem* 1997; **272**: 9609–9612.
18. Hannun YA, Linardic CM. Sphingolipid breakdown products: anti-proliferative and tumor-supressor lipids. *Biochem Biophys Acta* 1993; **1154**: 223–236.
19. Augé N, Andrieux N, Nègre-Salvayre A, Thiers JC, Lavade T, Salvayre R. The sphingomyelin-ceramide signalling pathway is involved in oxidized low density lipoprotein-induced cell proliferation. *J Biol Chem* 1996; **271**: 19251–19255.
20. Kimura S, Kawa S, Ruan F, Nisar M, Sodahira Y, Hakomori SI, Igarashi Y. Effect of sphingosine and its N-methyl derivatives on oxidative burst, phagokinetic activity, and trans-endothelial migration of human neutrophils. *Biochem Pharmacol* 1992; **44**: 1585–1595.
21. Mason RP, Hanna PM, Burkitt MJ, Kadiiska MB. Detection of oxygen-derived radicals in biological systems using electron spin resonance. *Experimental Health Perspectives* 1994; **102**: 33–36.
22. Rosen GM, Pou S, Ramos CL, Cohen MS, Britigan BE. Free radicals and phagocytic cells. *Faseb J* 1995; **9**: 200–209.
23. Evans CA. Spin trapping. *Aldrichimica Acta* 1979; **12**(2): 23–30.
24. Janzen EG. Spin trapping. *Acta Chem Res* 1979; **4**: 31–41.
25. Rosen GM, Britigan BE, Cohen MS, Ellington SP, Barber MJ. Detection of phagocyte-derived free radical with spin trapping techniques: effect of temperature and cellular metabolism. *Biochimica Biophysica Acta* 1988; **969**: 236–241.
26. Green MJ, Hill HAO. Chemistry of dioxygen. *Methods in Enzymology* 1984; **105**: 3–22.
27. Borgeat P, Samuelson B. Arachidonic acid metabolism in polymorphonuclear leukocytes: effects of ionophore A23187. *Proc Natl Sci USA* 1979; **76**: 2148–2152.
28. Easmon CSF, Cole PJ, Williams AJ, Hastings M. The measurement of opsonic and phagocytic function by luminol-dependent chemiluminescence. *Immunology* 1980; **41**: 67–72.
29. Britigan BE, Rosen GM, Chai Y, Cohen MS. Do human neutrophils make hydroxyl radical? *J Biol Chem* 1986; **261**: 17026–17032.
30. Britigan BE, Coffman TJ, Buettner GR. Spin trapping evidence for the lack of significant hydroxyl radical production during the respiration burst of human phagocytes using a spin adduct resistant to superoxide-mediated destruction. *J Biol Chem* 1990; **265**: 2650–2656.
31. Lai CS, Piette LH. Hydroxyl radical production involved in lipid peroxidation of rat liver microsomes. *Biochem Biophys Res Commun* 1977; **78**: 51–59.
32. Finkelstein E, Rosen GM, Rauckman EJ. Spin trapping of superoxide and hydroxyl radical: practical aspects. *Arch Biochem Biophys* 1980; **200**: 1–16.
33. Lee TC, Ou MC, Shinozaki K, Malone B, Snyder. Biosynthesis of N-acetylsphingosine by platelet-activating factor: sphingosine CoA-independent transacetylase in HL-60 cells. *J Biol Chem* 1996; **271**: 209–217.
34. Rothstein JL, Lint TF, Schreiber H. Tumor necrosis factor/cachectin. Induction of hemorrhagic necrosis in normal tissue requires the fifth component of complement (C5). *J Exp Med* 1988; **168**: 2007–2021.
35. Fuortes M, Jin W, Nathan C. Ceramide selectively inhibits early events in the response of human neutrophils to tumor necrosis factor. *J Leukoc Biol* 1996; **59**: 451–460.
36. Hannun YA. The sphingomyelin cycle and the second messenger function of ceramide. *J Biol Chem* 1994; **269**: 3125–3128.
37. Chao CP, Laulederkind SFJ, Ballou LR. Sphingosine-mediated phosphatidylinositol metabolism and calcium mobilization. *J Biol Chem* 1994; **269**: 5849–5856.
38. Wilson E, Olcott MC, Bell RM Jr, Merrill AH Jr, Lambeth JD. Inhibition of the oxidative burst in human neutrophils by sphingoid long-chain bases. *J Biol Chem* 1986; **261**: 12616–12623.
39. Samuni A, Black CDV, Murali-Krishna C, Malech HL, Bernstein EF, Russo A. Hydroxyl radical production by stimulating neutrophils reappraised. *J Biol Chem* 1988; **263**: 13797–13801.
40. Sasaki J, Yamaguchi M, Satoshi S, Yamane H, Okumara N, Ishibashi S. Sphingosine inhibition of NADPH oxidase activation in a cell-free system. *J Biochem* 1996; **120**: 705–709.

ACKNOWLEDGEMENTS. This work was supported by the FRSM (fund for Medical Scientific Research—Belgium) grant no. 3.4556.95.

The authors gratefully thank Dr G. Hartstein for his assistance with the English translation of this paper. They are also indebted to Ms Y. Goutman for excellent technical assistance.

**Received 15 February 1997; accepted in revised form 25 June 1997**
